# Digit salvage therapy with integrated Chinese and Western medicine: report of case series

**DOI:** 10.3389/fmed.2025.1656640

**Published:** 2025-11-06

**Authors:** Jiaming Cui, Hanbo Wang, Yanlong Yao, Junwei Zong, Ying Gong, Ran Sun, Mingzhi Song

**Affiliations:** 1 School of Basic Medical Sciences, Shenzhen University, Shenzhen, Guangdong, China; 2 Department of Biomedical Engineering, Stony Brook University, Stony Brook, New York, United States; 3 Department of Burn and Wound, Dalian Fourth People's Hospital, Dalian, Liaoning, China; 4 Department of Orthopaedics, The First Affiliated Hospital of Dalian Medical University, Dalian, Liaoning, China; 5 Department of Nursing, The First Affiliated Hospital of Dalian Medical University, Dalian, Liaoning, China; 6 Operating Room, The First Affiliated Hospital of Dalian Medical University, Dalian, Liaoning, China; 7 Institute of Translational Medicine, Jining Medical University, Rizhao, Shandong, China; 8 Department of Orthopedics, Affiliated Hospital of Jining Medical University, Jining, Shangdong, China; 9 Department of Orthopaedic Surgery, Shanghai Ninth People’s Hospital, Shanghai Jiao Tong University School of Medicine, Shanghai, China

**Keywords:** digit salvage therapy, tissue necrosis defect, integrated Chinese and Western medicine, NPWT, MEBO

## Abstract

**Background:**

Finger or toe trauma is a common orthopedic disease. However, the digit salvage treatment method for patients with severe tissue necrosis or infection is very limited.

**Methods:**

Five retrospective cases with typical lesions received our integrated traditional Chinese and Western medicine digit salvage therapy. These cases were with necrosis and defect or osteomyelitis. According to the patient’s different lesion characteristics, we carried out the integrated treatment of traditional Chinese and Western medicine including nibbling debridement, negative pressure wound treatment (NPWT), moisture burn ointment (MEBO), rehabilitation exercise and common surgical techniques.

**Results:**

We considered that precarious alive tissue (PAT) in the residual tissue of the lesion area was very important, especially for promoting tissue regeneration and repairing defects. By nibbling debridement, PAT can be accurately identified. NPWT could protect wound, control infection and fully absorpt the effusion. MEBO was used for dressing changes. While rehabilitation exercise is very important to promote the function recovery. Additionally, bone drilling, skin-grafting and immobilization were also used in different cases. The affected finger or toe healed completely during 1.5–18 months. All patients restored the movement function of the adjacent joints in the lesion area. There was no recurrence of infection or complaint of pain.

**Conclusion:**

Therefore, traditional Chinese and Western medicine digit salvage therapy may offer an alternative to amputation in select cases. However, further research with larger sample sizes and controlled trials are still needed before wide application of this therapy.

## Introduction

Finger or toe trauma is a common orthopedic disease, which can easily lead to tissue necrosis defect and deep infection. In the United States, 45,000 fingers are amputated per year with an incidence rate of 7.5/100,000 people (1). The incidence of finger amputation was bimodal. Young children and the elderly are most at risk (1, 2). Outstanding contributions of hand microsurgery and plastic surgery in finger reconstruction, flap repair, finger replantation and other work are very important (3–5). These continuously improved surgical techniques have played an important role in finger preservation, functional reconstruction, sensory recovery, and shape remodeling. However, serious tissue injury or infection to the finger or toe usually results in amputation (6, 7). In many cases, the decision to amputate is influenced by clinical judgment favoring quickly getting rid of infection and shortening the healing time. Generally, smashed injuries and infections with complete destruction of bone make it impossible to preserve digits. This may lead to decision of direct amputation without any attempt of salvage treatment. The true challenge is the borderline case. If we amputate too soon, we may forfeit a digit that could have recovered. If we persevere with salvage too long, infection and necrosis may progress. This requires us to accumulate more clinical treatment experience to accurately determine the possibility of salvage. Accordingly, we need to offer a feasible, clinically workable pathway in borderline cases. In these cases, the status of precarious alive tissue (PAT) acts as a key determinant of treatment. PAT usually appears at the edge of the wound and is a borderline tissue between necrotic tissue and healthy tissue. It is a significant indicator of the range of debridement.

Digit preservation even if it loses some function can at least provide appearance and psychological comfort (8). Unlike fresh digit injuries, severe post-traumatic tissue necrosis lacks the histological basis for replantation or flap surgery, and bacterial infection can lead to surgical failure. The rapid development of wound treatment can give patients hope of saving their digits. First, nibbling debridement is a surgical technique that can effectively promote tissue regeneration while removing necrotic tissue (9). It is especially good for digits with very little tissue volume. Then, the emergence of negative pressure wound treatment (NPWT) technology provides a possibility for adjusting wound microenvironment, controlling infection and accelerating granulation growth (10–12). Traditional Chinese Medicine (TCM) ointment has unique advantages of both clearing heat and detoxication and removing saprophytic muscle in repairing refractory infection wounds (13–15). Among them, the representative medicine is a moist burn ointment (MEBO) containing *β*-sitosterol, baicalin, and berberine as active ingredients in a base of beeswax and sesame (16). Current research indicates that MEBO mainly exerts a promoting effect on tissue regeneration by stimulating angiogenesis, optimizing the types of lesion microorganisms, and accelerating re-epithelialization (17–19).

Using the above three main techniques to give full play to the treatment characteristics of integrated Chinese and western medicine, we have already treated a small number of clinical cases and selected to share in the case series format. The relevant data of the limited case treatment was hoped to be the important foundation for conducting the further controlled trials.

This case series evaluated the possibility of achieving digit salvage through integrated Chinese and western medicine treatment (light bite debridement, NPWT and MEBO) for patients with severe tissue necrosis or infection.

## Case presentation

### Case 1

A 12-year-old female student came to the orthopedics clinic accompanied by her parents. She presented with swelling, pain and dislocation deformity in the left index finger.

Approximately 1 month prior to seeking treatment at our hospital, she underwent debridement, nail bed sutures and antibiotic therapy at the primary hospital. Her left index finger was accidentally crushed by a desk at the school. Bleeding, pain, and limited function were the main symptoms. The patient was sent to the nearest clinic for debridement and nail bed sutures. Although the patient received debridement and suture as well as antibiotic therapy, the gradual emergence of eschar and purulent exudation of the nail bed caused concern to her parents. After that, the patient went to the hand microsurgery department of several hospitals, the exact same treatment plan for finger amputation was given. It seemed impossible to preserve the finger.

**Figure 1:**
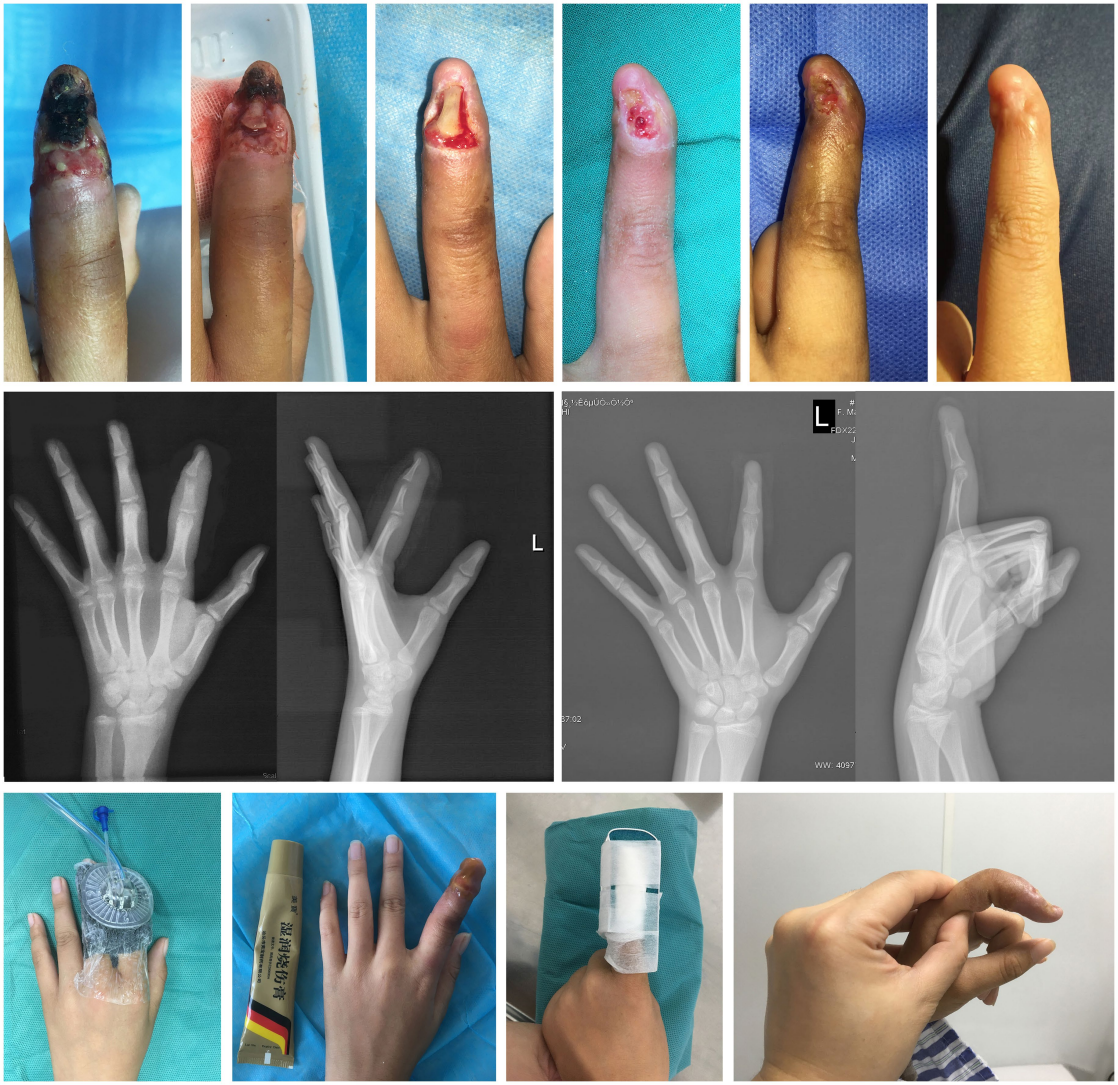
Case 1. The healing process, imaging findings and main treatment methods for the left index finger.

By physical examination, we found the affected finger was significantly red and swollen accompanied by purulent discharge. There was limited movement and dislocation deformity of the distal interphalangeal joint. The radiograph showed dislocation of interphalangeal joint and destruction of phalanx. The results of culture indicated *Staphylococcus aureus*. The patient was in good general condition and had no history of other diseases. The diagnosis included nail bed necrosis, extensive soft tissue defects, phalange exposed, rupture of extensor tendon, dislocation of the interphalangeal joint, and suppurative infection of the interphalangeal joint from an injury (Figure 1).

In order to ensure the patient’s study time at school, we developed a finger salvage therapy plan. In the course of three local anesthesia surgeries, nibbling debridement were finely carried out, and then NPWT was selected as the main treatment. The interval between surgeries was 3–5 days. During the non-surgical treatment period, MEBO was administered topically twice a day. The radiograph was taken regularly and wound secretions were cultured. When the result of the bacterial culture turned negative, the wound was stabilized and the osteomyelitis was controlled. We selected bone drilling to gradually stimulate the granulation tissue from the pulp cavity and gradually cover the entire wound. In the middle and later stages of treatment, the remaining phalanges showed obvious positional shifts. Therefore, the aluminum plate supported to correct the deformity.

The finger was finally saved and presented a satisfactory appearance, and functional compensation was achieved via rehabilitation exercises of the adjacent joint. The patient resumed school activities without pain, was satisfied with the finger’s appearance, and expressed relief and confidence. The range of motion (ROM) of the proximal interphalangeal joint was 0° when extended and 90° when flexed. The visual analogue scale (VAS) was 1. The treatment lasted a year and a half.

### Case 2

A 38-year-old male zipper factory worker came to the orthopedics clinic due to work-related injuries. His chief complaint was a shriveled right index finger, changes in skin color, numbness and limited movement.

Five weeks ago, his right index finger was strapped while he was working, and then there was no skin rupture or bad peripheral blood supply, only skin paresthesia in the damaged area. The emergency surgeon recommended observation without special disposal. After that, the affected finger gradually developed skin necrosis and limited joint movement. The orthopedic clinics of the many hospitals he visited tend to amputate his finger. So the patients come to our clinic for treatment.

**Figure 2:**
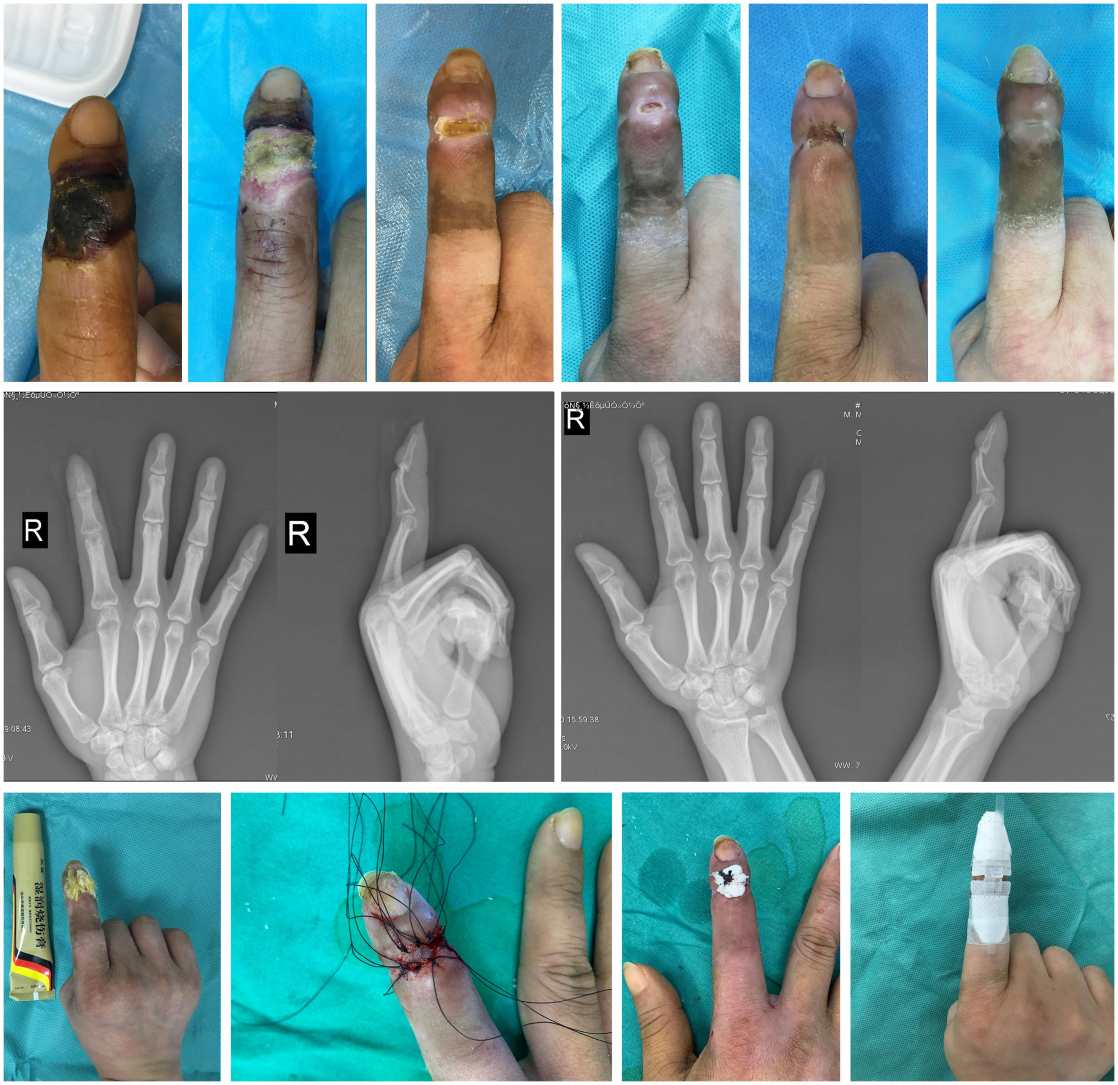
Case 2. The healing process, imaging findings and main treatment methods for the right index finger.

The main manifestations we found were eschar changes in the skin of the distal interphalangeal joint of the right index finger, limited movement, weakened interphalangeal sensation, and palpable bilateral vascular pulsation at the proximal end. X-ray examination showed no obvious abnormalities. The final diagnosis was full-thickness necrosis of the skin and soft tissue at the distal interphalangeal joint of the right index finger (Figure 2).

We first performed a debridement to determine the condition of his finger preservation. The full-thickness skin defect on the back was found with the extensor tendon insertion exposed. We decided to perform finger salvage. Nibbling debridement combined with NPWT was selected to gradually remove the clearly dead tissue. MEBO was administered topically for wound treatment during each debridement interval (3–5 days). After two and a half months, all the tissues of wound edge were new, but the growth of the tissues was hindered by base of distal phalanges. We use the rongeur to bite part of the bone. The wound grew slowly, and free skin grafts were performed after radiographs showed the presence of new tissue on the finger bone surface 2 months later. Then, the patient was continued to administer MEBO topically (twice a day) and prescribed rehabilitation exercises of other small joints until the wound was completely healed. Finally, he returned to work, reported improved sensation and felt the staged care was worth the time. ROM of the proximal interphalangeal joint was 0° when extended and 90° when flexed. VAS was 0. The total duration of treatment used to preserve the finger was 1 year.

### Case 3

A 58-year-old woman complained with swelling and pain of the right middle finger. She was a barbecue restaurant worker. Accompanied by the boss, she came to the hospital for diagnosis and treatment.

Two months ago, the patient was punctured by a bamboo skewer on the palmar side of her right middle finger while cleaning, but only underwent a simple disinfection treatment. She then went to a hospital for anti-infective treatment due to an acute infection such as redness and swelling. Within a month and a half, the patient was treated with three different antibiotics, and although the symptoms were less severe, the radiograph indicated osteomyelitis. Her doctor warned her that the infection would be difficult to treat and there was a risk of amputation. In order to seek better treatment and preserve her finger as much as possible, she came to our team for help.

**Figure 3:**
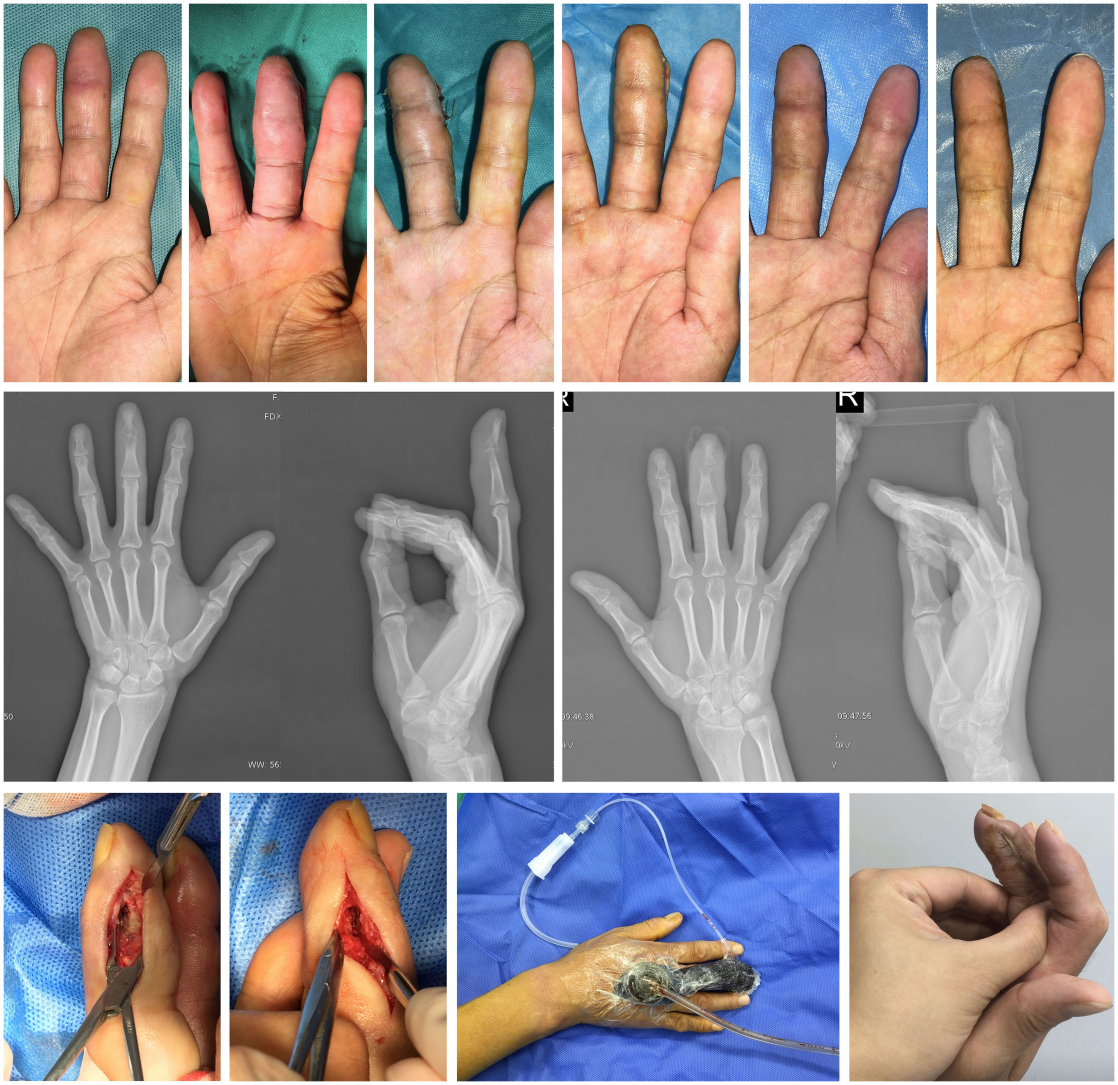
Case 3. The healing process, imaging findings and main treatment methods for the right middle finger.

Obvious swelling, tenderness and limited movement are the main findings of physical examination. Based on the medical history, physical examination and imaging results, the diagnosis was purulent distal interphalangeal arthritis of the right middle finger (Figure 3).

After assessing the overall condition of the affected finger, we carried out finger salvage treatment. Incision exploration and fine debridement were the completed in the first operation. The distal interphalangeal joint was severely eroded by infection with dead bone formation and accumulation of most of the deep flexor tendon insertion was destroyed. The retained secretions were sent for bacterial culture and showed to be *Bacillus aeruginosa*. A rinsing tube was placed inside the incision to rinse daily with normal saline, and the NPWT sponge was selected for adequate drainage from the other side. After three operations (10 days), the tissue inside the incision was fresh, the dead bone was basically cleared, and both incisions were sutured. In the later stage of incision management, MEBO was administered topically twice a day. In addition, the patient was prescribed rehabilitation exercises of the adjacent joints. ROM of the metacarpophalangeal joint was 20° when extended and 60° when flexed. VAS was 1. Over a period of 2 months, the patient’s right middle finger was successfully preserved. She regained functional grip with minimal pain, accepted mild residual stiffness, and felt grateful to avoid amputation.

### Case 4

A 51-year-old female worker in a garment factory came to the clinic with subungual abscess and persistent pain of the left index finger.

A month earlier, while working in a garment factory, the patient’s left index finger nail was stabbed by a sewing machine. At that time, the patient only received a simple disinfection. She later had to seek medical help because of a significant swelling pain under her nail. In this case, her first visit to the hospital recommended fingertip amputation, as radiograph revealed significant bone damage and a large amount of pus under the nail, indicating a serious infection. But the patient was more eager to preserve the finger, so she switched to our hospitals to seek help.

**Figure 4:**
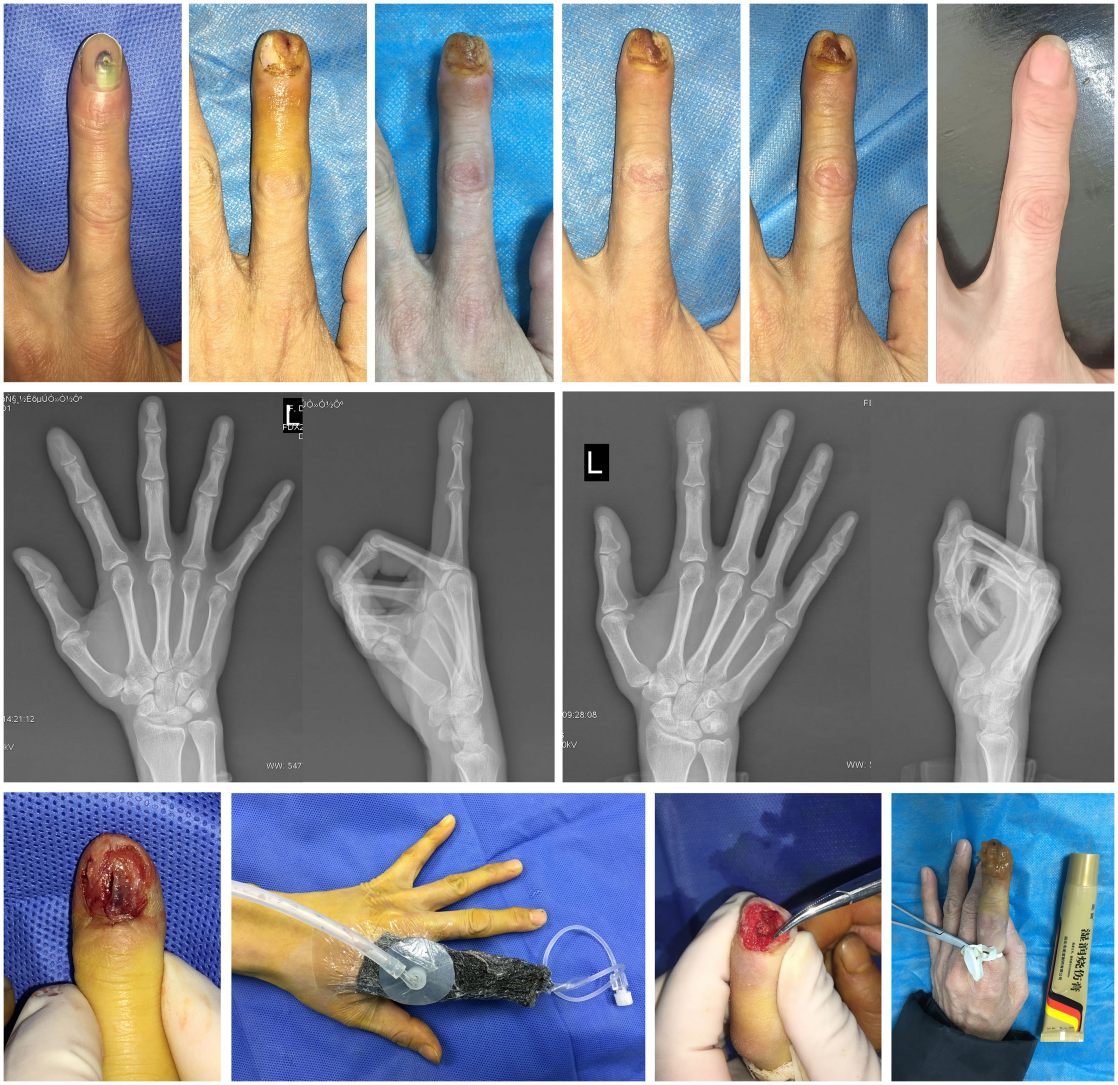
Case 4. The healing process, imaging findings and main treatment methods for the left index finger.

Physical examination revealed obvious redness and swelling at the tip of the left index finger, pus accumulation under the nail, paraesthesia of skin, nail tenderness, and no impact on the range of motion of each joint. Taking into account the imaging results, the diagnosis was confirmed as a phalangeal abscess (Figure 4).

Our finger salvage treatment plan was still based on debridement exploration. A bone defect has formed with a small amount of dead bone surrounded by old inflammatory granulation tissue. Fine removal of necrotic tissue and adequate drainage was gave. NPWT with the rinsing tube helps patients to relieve local pressure, remove undesirable fluid and promote tissue regeneration. The results of pus culture showed that *Staphylococcus aureus* infection. When all the lesion tissue turn fresh, MEBO was administered topically (twice a day) until complete healing. With the help of rehabilitation exercises guidance, the full range of motion of the affected finger was also preserved. She reported full motion, no pain, and high satisfaction with the cosmetic outcome. Because of the debridement principle of preserving as much viable tissue as possible, most of the nail bed is preserved and the final appearance is very desirable. ROM of the distal interphalangeal joint was 0° when extended and 90° when flexed. VAS was 0. The total duration of treatment with preserved fingers was 6 weeks.

### Case 5

A 59-year-old male physical laborer was sent to our orthopedic emergency department, because a wall collapse hitted the dorsum of his right foot directly. His mainly complained of pain, bleeding and limited movement of the right foot. The laceration of all the toe webbed skin and the degloving injury of the dorsal skin indicated that the patient was at high risk for tissue necrosis. The radiograph showed multiple fracture of the right foot. The patient was admitted to the hospital with a diagnosis of fractures of the 2nd, 3rd, 4th and 5th metatarsal bones accompanied by degloving injury of the dorsal foot skin. In emergency surgery, our goal is to use Kirschner’s needle to fix the fracture in the simplest way possible, and to reattach or suture the skin and soft areas as much as possible. We performed the patient plaster immobilization, medical infrared physiotherapy, and MEBO local application. The surgery went well, but the skin on the back of the foot and the third toe showed signs of necrosis 2 weeks after surgery. A month after surgery, the necrotic tissue was well defined. After discussion within the department, most doctors thought that toe amputation was an ideal choice, but our treatment team hoped to use the previous experience of finger preservation to try toe preservation.

**Figure 5:**
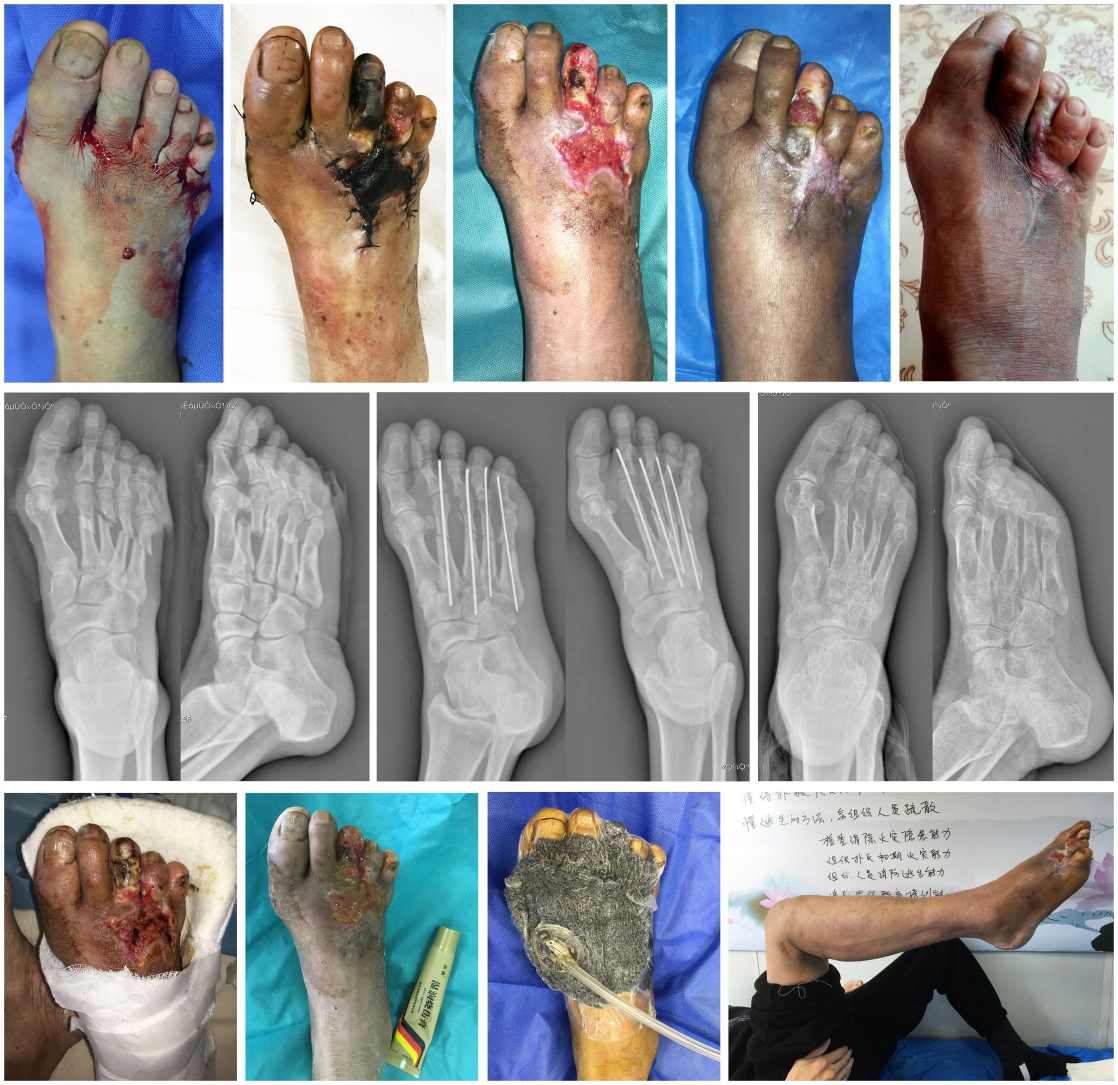
Case 5. The healing process, imaging findings and main treatment methods for the third toe of right foot.

We conducted another physical examination on the patient and found that the top of the right foot and the fourth toe were shriveled, there was an abnormal sensation in the local skin, and the movement of all joints except the big toe was restricted. Therefore, the supplementary diagnosis is gangrene of the skin on the dorsal part of the right foot and the third toe, as well as postoperative conditions of the 2nd, 3rd, 4th, and 5th metatarsals (Figure 5).

The treatment plan was still to preserve as much tissue as possible after fine debridement and then treated by NPWT, which was conducted three times in total (once every 5 days). When the surrounding new tissue cannot repair the central defect, bone drilling was performed. The phalanx was completely wrapped in soft tissue by local application of MEBO (twice a day). Finally, the patient achieved toe salvage. The last radiograph showed that the four metatarsal fractures had healed. We also prescribed rehabilitation exercises to the patient, including hip, knee, ankle and foot. ROM of the metatarsophalangeal joint was 30° when extended and 20° when flexed. VAS was 0. There was no limb deficiency outcome after 4 months of treatment. He returned to daily ambulation and work shoes, accepted the cosmetic result, and felt relieved about toe salvage.

## Discussion

Patients with digits trauma are very common, and patients with complication of severe soft tissue injury and deep infection are not rare, especially in the elderly (20). Tissue necrosis and infection can lead to an increased risk of digit amputation. Early amputation is fast and reliable. It provides source control and predictable closure. It also sacrifices length and sensate skin and ends fine pinch. Patients may face stump revision, phantom pain, and prosthetic adaptation. There are still few reports on surgical methods of minimal intervention for limb preservation. In fact, digital osteomyelitis has been an expensive and morbid disease. Aggressive surgical debridement/digit amputation and selected use of arterial bypass should improve wound healing and limb salvage, respectively (21). In contrast, antibiotic therapy alone is associated with decreased wound healing and limb salvage (22). Flap is the most commonly used method for treating soft tissue defects in clinical practice and has also become a key choice for digital salvage (23). It is highly regarded for its rapid postoperative recovery and the fact that the local repaired tissue structure is closer to the recipient area. However, flap surgery has very high requirements for blood supply to both the recipient and donor areas, and it also demands strict technical skills from the surgeon. Flap reconstruction adds donor-site morbidity, resource demands, and a higher risk of failure in contaminated fields. Additionally, there is a certain probability of necrosis in the flap. In conclusion, developing simple and effective digit salvage methods is of vital importance for clinical treatment.

Due to the rapid development of wound repair technique, the clinical ability to preserve injured or infected tissue is improving. New technologies and ideas are also constantly updated. Our approach is to integrate new wound treatment technologies to address the limitations of the conventional care methods. NPWT, in particular, is of great help in promoting wound healing (24). It also has a good effect on bone repair (25). They are believed to hasten wound healing by maintaining a moist environment, removing wound exudates, increasing local blood flow, increasing granulation tissue formation, applying mechanical pressure to promote wound closure, and reducing bacterial loads in the wound (26). TCM also has its unique features for wound therapy, including clear therapeutic effect, user-friendly operation, no special requirements for medical devices, lower treatment cost (27). Nibbling debridement is an old technique from surgery of TCM. It requires operations similar to biting off little by little as a silkworm. During the treatment of these cases, we usually use ophthalmic scissors in combination with ophthalmic tweezers for the operation. The scope and depth of debridement should be such that there is no bleeding. In this process, the tissue that has been identified as dead is removed, and tissue that has not been completely necrotic is preserved to the greatest extent possible. This technique has been applied in many fields, such as wounds, necrotizing fasciitis, diabetic foot, etc. (9, 28, 29). Nibbling debridement could be involved in improving the blood flow changes and inflammatory cell infiltration in animal limb ischemia models, reducing the degree of collagen deposition and vascular fibrosis (30). MEBO wraps the wound with beeswax, providing a moist environment conducive to promoting tissue healing as well as achieves the effect of promoting wound healing by adjusting the changes in microbial populations in the microenvironment (18). Based on the above discussion, MEBO and NPWT share similarities in improving the microenvironment of wounds and promoting tissue healing. Chinese herbal medicine and negative pressure wound therapy have been proved with increasing the blood supply in the affected area (31). For the cases of severe necrosis of the limb tissue, the limb preservation method of integrated Chinese and Western medicine has been proven to work (32, 33). In this study, the cases we mentioned were all achieved by treatment of integrated traditional Chinese and Western medicine.

Of the five patients, two were men and three were women. There were four workers and one junior middle school student. The age range was 12–59 years. The common feature of the patients was that they all had a very clear history of trauma. The main manifestations of the lesion area were infection and tissue necrosis. After wound exposure, phalanges, interphalangeal joints, tendons, nail beds, and residual tissue can be directly observed. Under the treatment concept of integrated Chinese and Western medicine, we choose reasonable treatment methods according to the actual situation of patients. In treatment, the most used method is nibbling debridement, NPWT, MEBO and rehabilitation. Eventually, the patients were able to save their digits and return to normal life. In our five-case series, the time to durable wound closure averaged 30 weeks (range 6–72). Cases with relatively simple deep infection (Cases 3–4) healed within 6–8 weeks, whereas those with severe soft-tissue necrosis and tendon/joint or bony involvement (Cases 1–2) required 48–72 weeks (Table 1). While conventional pathways may shorten time-to-closure, our staged, digit-preserving strategy intentionally traded time for function. Compared with the commonly used digit salvage therapy methods at present, the approach we proposed has certain advantages, including low surgical difficulty, flexible treatment plans and good therapeutic effect.

**Table 1:** Patient information.

Case number	Sex	Age (years)	Pathogenesis	Main disease type	Lesion location	Exposed structures	Duration of treatment (months)	Main therapeutic method
1	W	12	Injury caused by heavy objects	Infection and tissue necrosis	Distal interphalangeal joint and distal section of left indicative finger	Distal phalangeal bone, interphalangeal joint, residual tendon at the insertion of extensor tendon	18	Nibbling debridement, NPWT, MEBO, immobilization and rehabilitation
2	M	38	Strapping wound	Tissue necrosis	Distal interphalangeal joint of the right index finger	Interphalangeal joint and extensor tendons	12	Nibbling debridement, NPWT, MEBO, skin graft and rehabilitation
3	W	58	Stab wound	Infection	Distal interphalangeal joint of right middle finger	Interphalangeal joint	2	Incision and drainage, nibbling debridement, NPWT, MEBO and rehabilitation
4	W	51	Stab wound	Infection	Distal section of left index finger	Distal phalangeal bone and nail bed	1.5	Incision and drainage, nibbling debridement, NPWT, MEBO and rehabilitation
5	M	59	Injury caused by heavy objects	Tissue necrosis	Right third toe and foot dorsalis	Interphalangeal joint, extensor toe tendon and distal phalanx	4	Nibbling debridement, NPWT, MEBO, immobilization and rehabilitation

W: woman; M: man; NPWT: negative pressure wound treatment; MEBO: moisture burn ointment.

The contents of the digits are small, and much of the space is occupied by the phalanges and tendons, so that tissue regeneration and repair in this area is very difficult. As the prime movers of tissue repair, blood vessels and residual tissues are crucial factors. Actually, the key to our treatment is to induce these tissue repair through the methods of integrated traditional Chinese and Western medicine. For lesions of severe injury or infection, there is a special tissue between normal healthy tissue and necrotic tissue, which is called PAT. PAT has functionally impaired, but still survive. As early as the end of the last century, researchers had described a special tissue in burn wounds, which was similar to PAT (34). Interestingly, PAT was found in our clinical work, almost in all wounds, often attached to the surface of the wound. Therefore, PAT is critical for tissue repair and reconstruction. However, in the basic principles of surgical debridement, thorough debridement is essential. As a large amount of tissue (necrotic tissue, PAT, normal tissue) is removed, the bleeding of the wound has became a marker for the surgeon to determine to stop debridement. When we became aware of the presence of PAT, we optimized the principle of debridement to remove as much the real dead tissue as possible and stop debridement as soon as oozing of the blood was detected. During debridement and epluchage, NPWT treatment can prevent the exposure of deep bone, tendon and other tissues (35). In digit salvage treatment, we generally choose 3–5 days as the duration of each NPWT. And the selected negative pressure value is 125 mmHg.

Often, after a severe injury to the digit, further secondary necrosis of the tissue occurs. Multiple nibbling debridement can be used with or without postoperative NPWT until the dividing line between necrotic tissue and normal tissue appears. It should be noted that drainage of the deep diseased tissue must be ensured to be unobstructed. The surgical procedure should be more careful at this time as the PAT becomes more pronounced. Nibbling debridement provides an important means for PAT retention. Its advantages are more accurate debridement procedures and a surgical field of view that facilitates observation of tissue status. In the negative pressure environment created by NPWT, efficient drainage will effectively inhibit the proliferation of antibacterial bacteria and reduce the further destruction of residual tissue by inflammatory exudate. NPWT should be given priority for patients with severe infection. Moreover, NPWT has a strong ability to promote tissue regeneration, and this advantage of accelerating wound closure is obvious. As a classic and effective TCM ointment, MEBO can promote the liquefaction of necrotic tissue and stimulate the new vitality of residual tissue (13). For burn wounds, the safety of dressing change with MEBO is significantly better than that with iodophor and silver ion drug (36). MEBO has also used as part of a combination of traditional Chinese and Western medicine to treat diabetic foot ulcers (37). For the above five cases, we adopted the method of local application of MEBO twice a day for treatment. The dosage of the ointment used is the required amount to completely cover the area of tissue defect. In the case of complete bone exposure, drilling can induce the growth of new tissue and achieve the effect of tissue repair (38). Here, we used this technique to repair bone exposed area for case 1 and case 5. The soft tissue in the dorsal digital area is thin, especially the nail bed, which is only 2 mm thick. It is difficult to repair soft tissue defects by growth of surrounding tissues. Under normal circumstances, the repair method is mainly flap operation. However, flap operation is not appropriate for chronic or infectious wounds after trauma. This bone stimulation method effectively stimulated the growth of mesenchymal stem cells in the bone marrow cavity to form granulation to wrap the exposed area. Therefore, patients recommended for bone drilling should be those with thin wounds and poor blood supply areas where there is no possibility of flap surgery repair. In addition, rehabilitation exercise also needs to be emphasized in the treatment, because in addition to the affected interphalangeal joints, other joints should be early non-weight-bearing rehabilitation exercises. Specifically, the patients were required to actively perform active flexion and extension exercises of the adjacent joints of the lesion, with 10 sets each time and 10 repetitions in each set. The concept of early rehabilitation has been infused into all the cases of traditional Chinese and Western medicine. While treating the patient’s finger, the patient’s limb function is preserved to the greatest extent. In order to accelerate the rate of epithelialization, free skin graft can be selected before the wound is completely healed. Braces including the aluminum plate and plaster are necessary for patients combined with fractures or dislocations. For the upper extremities, patients are encouraged to perform flexion and extension of the interphalangeal and metacarpophalangeal joints other than the injured joint to compensate for the function of the injured joint. Patients with toe injury are asked to strengthen the active movement of the hip and knee joint, which can reduce the impact of immobilization of the affected limb and prevent the occurrence of deep vein thrombosis of the lower limb. Based on the above discussion, we completed these five representative digit-preserving therapy under the guidance of the treatment concept and program of integrated Chinese and western medicine. The complete clinical information and timeline have been listed in Table 2.

**Table 2:** Complete clinical information and timeline.

Case number	Occupation	Mechanism	Outside care	Pathogen	Family/psychosocial status	Final ROM	Timeline
1	Student	Crush by the school desk	Debridement + nail-bed sutures; antibiotics	*Staphylococcus aureus*	Accompanied by parents; schooling prioritized	PIP 0°–90°	Trauma → outside debridement/sutures/antibiotics → presentation (~1 m) → serial debridement ×3 (q3–5 d) + NPWT → phalangeal drilling → deformity correction → rehabilitation → closure/end 18 m
2	Zipper factory worker	Work-related strap injury	Observation; multiple amputation recommendations	–	Occupational injury; preservation prioritized	PIP 0°–90°	Work injury → observation elsewhere (5 w) → presentation → debridement + NPWT → bone trim → free skin graft → rehabilitation → closure/end 12 m
3	Barbecue restaurant worker	Bamboo skewer puncture	Disinfection; three antibiotic courses; osteomyelitis on X-ray	*Bacillus aeruginosa*	Accompanied by employer; manual work demands	MCP 20°–60°	Puncture → outside antibiotics (6 w) → presentation (~2 m) → incision exploration + debridement → rinse catheter + NPWT → closure (8 w) → rehabilitation
4	Garment factory worker	Nail puncture by sewing machine	Initial disinfection; amputation advised elsewhere	*Staphylococcus aureus*	Desire for cosmesis and function; job-related needs	DIP 0°–90°	Puncture → swelling/pain → presentation (~1 m) → debridement + drainage → NPWT + rinse → MEBO → rehabilitation → closure (6 w)
5	Manual laborer	Wall collapse (crush)	K-wire fixation; skin reattachment; plaster; IR therapy; MEBO	–	Return-to-work considerations	MTP 30° ext./20° flex	Wall collapse → emergency immobilization/suturing → necrosis (2–4 w) → fine debridement → NPWT ×3 (q5 d) → phalangeal drilling → MEBO → rehabilitation → closure (16 w)

IR: infrared; NPWT: negative pressure wound treatment; MEBO: moisture burn ointment; ROM: range of motion; DIP: distal interphalangeal joint; PIP: proximal interphalangeal joint; MCP: metacarpophalangeal joint; MTP: metatarsophalangeal joint; ext/flex: extension/flexion; bid: twice daily; q3–5 d/q5 d: every 3–5 days/every 5 days; m: month(s); w: week(s); d: day(s).

Despite, in the treatment process of the above cases, there are still some limitations. This is a single center, retrospective case series with five patients and no control group. Selection bias is likely. Outcome assessment was not blinded. This kind of treatment with integrated Chinese and Western medicine needs enough patience and time. While our cases suggest feasibility, the 30-week average healing time underscores the need for comparative studies to assess efficiency versus standard care. The healing time of patients with simple deep infection is much shorter than that of patients with severe tissue necrosis. Therefore, the latter is more difficult to treat than the former. It generally takes more than 3 months for these patients to fully recover. It needs to be pointed out in particular that our proposed approach is suitable for patients with tissue necrosis and infection after initial treatment. For patients with acute trauma, primary surgical treatment such as debridement, skin grafting, skin flap, incision and drainage should be the first treatment. Under the background of the rapid development of artificial intelligence and surgical robot assisted technology, tissue damage repair treatment will certainly enter a new stage of finer and maximized patient benefits. The development of new drugs and materials is also important for improving the therapeutic effect. Collagen is a versatile structural biopolymer with self-assembly directed by extracellular cues, forming diverse hierarchical architectures that underpin tissue-specific functions. Using collagen-based dressings or collagen-mimetic scaffolds to reconstitute a provisional extracellular matrix could accelerate granulation and re-epithelialization in digit wounds (39). At present, the study of PAT is relatively few, and it is necessary to further study the mechanism of PAT production and promoting tissue repair. Additionally, the digit-preserving therapy methods listed by us are still limited, and more clinical practice is needed to explore the most effective digit-preserving therapy plan. Future studies should be multicenter and comparative. Such studies will test efficacy and cost-effectiveness and will refine selection and conversion criteria.

## Conclusion

Under the treatment concept of integrated Chinese and Western medicine, we preserved five cases of digit necrosis or infection caused by trauma. Methods used in treatment included nibbling debridement, NPWT, MEBO, rehabilitation exercise and common surgical techniques such as skin grafts and dressing changes. In the future, PAT will have profound significance in the research of the mechanism of tissue defect repair and the selection of seed cells for cell therapy. Our series of case reports aims to add new method about digit salvage therapy, so that these patients can achieve optimal therapeutic outcomes. This approach may benefit patients with digit necrosis or infection.

## Data Availability

The original contributions presented in the study are included in the article/supplementary material, further inquiries can be directed to the corresponding author/s.
